# Discrimination of English Vowel Contrasts in Chinese Listeners in Relation to L2-to-L1 Assimilation

**DOI:** 10.3390/bs15101420

**Published:** 2025-10-19

**Authors:** Youngja Nam

**Affiliations:** Centre for Creative Convergence Education, Hanyang University, 55 Hanyangdeahak-ro, Sangnok-gu, Ansan 15588, Republic of Korea; nature2024@hanyang.ac.kr

**Keywords:** Perceptual Assimilation Model-L2, assimilation overlap, overlap scores, goodness ratings, Chinese listeners, English vowels

## Abstract

The Perceptual Assimilation Model (PAM)-L2 framework posits that the discriminability of L2 speech contrasts can be predicted from how L2 phones are assimilated to L1 categories. This study examined how such assimilation types relate to variability in L2 vowel discrimination within the PAM-L2 framework, with particular attention to assimilation overlap. Chinese listeners were tested with six English vowel contrasts (/i-ɪ/, /e-ɛ/, /æ-ɛ/, /ɑ-ɔ/, /ɔ-ʌ/, /u-ʊ/) using an assimilation task with goodness ratings and an AXB discrimination task. The vowel contrasts formed three Uncategorized-Categorized and two Uncategorized-Uncategorized contrasts, with both types exhibiting either partial or complete overlap, along with one Category-Goodness contrast. Discrimination results showed that partial versus complete overlap accounted for some differences in discrimination accuracy and absence of overlap between dominant L1 response categories likely facilitated discrimination even when secondary categories overlapped. Large differences in perceived goodness appeared to facilitate discrimination for a vowel contrast with complete overlap. The results are discussed in particular relation to the PAM-L2 account of the assimilation overlap-discrimination relationship, and additionally how the influence of overlap may be modulated by category-goodness differences in contributing to variability in L2 vowel discrimination.

## 1. Introduction

Non-native speech contrasts are not uniformly difficult to discriminate, but rather their discriminability varies across contrasts. For example, English listeners with no exposure to Norwegian were highly accurate in discriminating the Norwegian vowel contrast /ki-kʉ/ (100%), but their accuracy dropped to 72.56% when discriminating another Norwegian vowel contrast /ki-ky/. One central framework for explaining this variability in non-native speech perception is the Perceptual Assimilation Model (PAM; Best, 1994, 1995) which accounts for how non-native speech sounds are perceived through the lens of native phonological categories in naïve listeners. Its extension, PAM-L2 (Best & Tyler, 2007), broadens the model to describe how second language (L2) listeners perceive L2 contrasts by taking into account the influence of native language (L1) and the development of L2 phonological knowledge. Another central framework is the Speech Learning Model (SLM; Flege, 1995) which provides an account of L2 speech learning with a focus on L2 production rather than L2 perception. Whereas the PAM-L2 proposes a typology of perceptual patterns for L2 speech contrasts and their respective discriminability, the SLM emphasizes the learnability of individual L2 phones. The present study specifically examines perceptual patterns for L2 contrasts. Therefore, PAM is the focal framework.

Within the PAM/PAM-L2 framework, contrasts involving less assimilation overlap are generally easier to discriminate than those with greater overlap (Faris et al., 2016, 2018). While numerous studies have tested these predictions in various L2 populations, relatively little research has examined Chinese listeners’ perception of English vowels from the perspective of assimilation overlap within the PAM/PAM-L2 framework. The present study addresses this gap by applying the PAM/PAM-L2 framework to examine Chinese listeners’ discrimination of English vowel contrasts, with particular attention to whether assimilation overlap helps explain their perceptual patterns.

### 1.1. Perceptual Assimilation Model-L2

In PAM-L2, an L2 phone is labeled as either categorized—if it matches a specific L1 category above a given threshold—or uncategorized—if no such L2-to-L1 match meets the threshold (Bundgaard-Nielsen et al., 2011; Tyler et al., 2014). PAM-L2 outlines a set of assimilation types that characterize how L2 phones are perceived in relation to L1 phonological categories. In this framework, Two-Category (TC) assimilation occurs when two L2 phones are categorized as two distinct L1 categories. TC-assimilated contrasts are expected to be easily discriminated. Single-Category (SC) assimilation occurs when both L2 phones are mapped onto the same L1 category with similar goodness-of-fit. SC-assimilated contrasts are expected to be difficult to discriminate. Category-Goodness (CG) assimilation refers to cases in which both L2 phones are categorized as the same L1 category but differ in how well they match it. CG contrasts are generally expected to be less challenging than SC contrasts and their discriminability depends on the degree of difference in category goodness between the two phones. In Uncategorized-Categorized (UC) assimilation, one phone is categorized as an L1 category, while the other is not assimilated to any L1 category. UC-assimilated contrasts are generally expected to be fairly good. Uncategorized-Uncategorized (UU) assimilation occurs when both L2 phones fail to be categorized, with discrimination accuracy varying depending on their phonetic distance and perceived similarity. Lastly, in Non-Assimilable (NA) assimilation, neither phone is perceived as speech and discrimination is expected to vary depending on their auditory distinctiveness.

More recent extensions to the model have introduced finer distinctions within the uncategorized response type. Faris et al. (2016) proposed three types of uncategorized responses: focalized, where an L2 phone shows above-chance mapping to a single L1 category; clustered, where multiple L1 categories are selected above chance; and dispersed, where no category is chosen consistently above chance. These distinctions help clarify how L2 phones that fall outside clear L1 categories are perceived. Faris et al. (2016) also introduced a three-level distinction in assimilation overlap—non-overlapping, partially overlapping, and completely overlapping—to account for variability in the discrimination of UC and UU contrasts. Discrimination was expected to follow a graded pattern, with non-overlapping contrasts yielding the highest accuracy, followed by partially and then completely overlapping contrasts. The influence of assimilation overlap was observed in the study of Faris et al. (2018) which examined Australian English listeners’ perception of Danish vowels, showing that the extent of phonological overlap tended to influence how well listeners discriminated the L2 vowel contrasts. Faris et al. (2018) examined Australian English listeners’ perception of Danish vowels to explore the role of phonological overlap in discrimination. Their findings showed that the extent of overlap influenced how accurately listeners discriminated UC- and UU-assimilated contrasts, generally supporting the PAM-based predictions.

### 1.2. Assimilation Overlap and L2 Vowel Discrimination

Many studies have examined the relationship between assimilation overlap and vowel discrimination in L2 listeners. One line of research has approached this by quantifying the degree of overlap using assimilation-based scores (Flege & MacKay, 2004; Levy, 2009). A key early study by Levy (2009) investigated English listeners’ perception of French vowels and found that contrasts with lower assimilation overlap were generally discriminated more accurately. In that study, overlap scores also tended to decline with increased L2 experience, suggesting that perceptual mappings may be restructured through exposure over time. These overlap scores are typically computed by identifying the proportion of shared L1 categories used to identify each member of a given L2 contrast. For example, if L2 phones X and Y are each mapped to multiple L1 categories, the score reflects the summed proportion of shared mappings.

Support for this association has also been provided in other L1-L2 pairings. Using this quantitative approach, Baigorri et al. (2019) examined Spanish-English bilinguals’ perception of English vowels and found that vowel contrasts with greater identification overlap tended to yield lower discrimination accuracy. Their findings provide additional evidence that overlap-based perceptual similarity constrains L2 discrimination, even in bilinguals with extensive exposure to both languages. These findings also suggest the relevance of assimilation overlap in shaping discrimination performance across different L1-L2 pairings.

Georgiou (2022) examined Cypriot Greek learners’ perception and production of the English contrast /ɪ-i:/ using discrimination and imitation tasks. His findings showed that higher identification overlap was associated with lower discrimination accuracy, and this pattern also extended to production: learners with greater perceptual overlap tended to produce the contrast less distinctly. These results suggest that assimilation overlap can constrain both L2 perception and production.

More recently, Fabra and Tyler (2023) examined English vowel discrimination in L1-Spanish-Catalan learners of English, applying both PAM-based assimilation overlap types (i.e., no, partial, and complete overlap) and overlap scores. Their findings showed that vowel contrasts with higher degrees of overlap generally tended to yield lower discrimination accuracy. For example, at the group level, the /i-ɪ/ contrast, classified as UC assimilation with no overlap, had a low overlap score (13.3%) and yielded high discrimination accuracy (97.8%), whereas the /ʌ-ɑ/ contrast, classified as UC assimilation with complete overlap, showed a high overlap score (83.0%) and relatively lower accuracy (67.2%). Group-level correlation analysis revealed a significant negative association between overlap scores and discrimination accuracy, supporting the view that assimilation overlap can shape L2 vowel discrimination, though this pattern was less consistent at the individual level.

### 1.3. The Present Study

The broad objective of the present study is to test key principles of the PAM-L2 framework by examining how assimilation patterns relate to L2 vowel discrimination. In particular, the study focuses on the role of assimilation overlap in shaping discrimination accuracy. While many studies have explored the impact of assimilation overlap in L2 speech perception, relatively few have examined Chinese listeners’ L2 vowel perception from this perspective. By analyzing both assimilation patterns and discrimination performance in this listener group, the study aims to contribute to the PAM-based literature on how overlap may influence L2 vowel perception across different L1-L2 pairings.

For Chinese listeners, prior work has examined perceptual patterns for a range of English vowel contrasts but only a few studies have reported both assimilation and discrimination. In an English-category identification task with synthesized vowels, X. Wang (2006) reported that Chinese listeners did not reliably form two-category distinctions for /i-ɪ/, /æ-ɛ/, or /u-ʊ/ (see also Hu et al., 2019; Y. Wang et al., 2022, for English-category identification). Jia et al. (2006) tested Chinese listeners’ discrimination of six English vowel contrasts (/u-ɑ/, /i-eɪ/, /æ-ɑ/, /i-ɪ/, /æ-ɛ/, /ɑ-ʌ/) and found that discrimination accuracy varied across contrasts. Sun and van Heuven (2007) examined English-to-Chinese mappings for 19 English vowels with Chinese listeners and observed that several vowels were most frequently mapped to the same Chinese vowel categories (e.g., /æ, ɑ, ɔ, ʌ/→Chinese /ɑ/). Lai (2010) provided both assimilation and discrimination data for seven English vowel contrasts with low- and high-proficiency Chinese listeners in Taiwan. Despite some differences in assimilation types, both groups showed a similar discrimination ordering, with /æ-ɛ/ the highest and /i-ɪ/ the lowest.

Motivated by the limited research on how assimilation relates to discrimination in Chinese listeners’ perception of English vowels within the PAM-L2 framework, the present study examined this relationship using both an assimilation task and an AXB discrimination task. Six English vowel contrasts were tested: /i-ɪ/, /e-ɛ/, /æ-ɛ/, /ɑ-ɔ/, /ɔ-ʌ/, and /u-ʊ/. To the author’s knowledge, this is the first study to investigate Chinese listeners’ discrimination of the /ɑ-ɔ/ and /ɔ-ʌ/ contrasts since no prior research has provided discrimination data on these vowels in this population.

Assimilation data were used to determine the perceptual mapping of each English vowel onto L1 categories and these patterns were then used to assess the degree of overlap for each vowel contrast. When a contrast was identified as UC or UU based on the assimilation results, it was further grouped as non-overlapping, partially overlapping, or completely overlapping following the PAM-L2 framework. In addition to these categorical overlap types, overlap scores were computed as a complementary tool to further describe the role of perceptual overlap (e.g., Levy, 2009; Baigorri et al., 2019).

Different studies have adopted various versions of the Chinese vowel system and the corresponding response options available to listeners varied. This variability makes it difficult to predict contrast-level assimilation patterns. Nevertheless, based on earlier findings (Sun & van Heuven, 2007), the following predictions can be made: /e-ɛ/ and /u-ʊ/ are expected to be non-overlapping, showing higher discrimination; and /i-ɪ/, /æ-ɛ/, /ɑ-ɔ/, and /ɔ-ʌ/ are expected to be overlapping, showing relatively lower to lowest discrimination depending on the degree of overlap.

## 2. Materials and Methods

### 2.1. Participants

Thirty-two Chinese-speaking adults (20 males and 12 females; mean age = 27.1 years, range = 22–35 years, SD = 3.8) participated. All were undergraduate or graduate students enrolled at universities in Seoul and were residing in Korea as international students at the time of testing. Length of residence in Korea ranged from 5 months to 8 years (mean = 3.8 years, SD = 1.9). According to Test of Proficiency in Korean (TOPIK) scores, 24 participants were advanced learners of Korean and 6 were intermediate learners. All participants were born and educated in China and had not resided outside the country prior to moving to Korea for their studies. Based on self-report, 24 participants indicated basic to lower-intermediate English proficiency and 6 reported upper-intermediate proficiency. None reported fluency in any other foreign language and none reported any hearing, speech, or language impairments. All participants received payment for their participation.

### 2.2. Stimuli

The stimuli consisted of ten English vowels produced in a /hVd/ context: heed (/i/), hid (/ɪ/), hayed (/e/), head (/ɛ/), had (/æ/), hod (/ɑ/), hawed (/ɔ/), hud (/ʌ/), hood (/ʊ/), and who’d (/u/). A 26-year-old female native speaker of Canadian English recorded the tokens in isolation using a Sennheiser microphone in a sound-attenuated booth. Recordings were made at a 44.1 kHz sampling rate using Praat software (Boersma & Weenink, 2021). The speaker produced each token multiple times and eight tokens per syllable were selected. Praat was used for editing and analyzing the tokens.

### 2.3. Procedure

Participants completed the experiment individually in a quiet place. Stimuli were presented and responses were recorded using the Paradigm Player software (Tagliaferri et al., 2010). Participants listened through headphones and the stimuli were presented at a comfortable listening level. As in the prior PAM-based studies (e.g., Nam et al., 2021; Tyler et al., 2014), the AXB discrimination task was administered before the identification task. The software automatically advanced to the next trial after each response. All on-screen instructions were presented in Chinese. The full experiment lasted approximately one hour per participant.

In the AXB discrimination task, participants were presented with four types of AXB trials: AAB, ABB, BAA, and BBA. Each trial presented triads of /h/-Vowel-/d/ syllables (e.g., /hid/-/hid/-/heed/) in which two vowels were the same and one vowel differed. Participants were asked to click “first” if the second vowel (X) matched the first vowel (A) or “third” if it matched the third vowel (B). The task began with one practice block, followed by eight test blocks. Each block consisted of 48 trials. Each of the six vowel contrasts included 16 trials per AXB type. Each AXB trial always featured physically different tokens for phonemically identical ones (e.g., A_1_A_2_B_1_). The inter-stimulus and inter-trial intervals were 1 s and 1.5 s, respectively. The order of the test blocks and the trial sequence within each block were randomized for each participant. Three self-paced breaks (2–5 min) were provided after the 2nd, 4th, and 6th blocks.

For the assimilation task, participants were asked to label the vowel in each /hVd/ syllable by selecting one of the six Chinese orthographic symbols displayed on the screen. The available options were /i, a, e, o, u, ü/, with each symbol aligned with its respective IPA vowel (/i, a, ɤ, o, u, y/) (Lee & Zee, 2003; Mou et al., 2018; Wu & Shih, 2009).

Following their assimilation response, participants evaluated how well the English vowel they heard matched their selected Chinese vowel category. This rating was made using a 5-point scale, with 1 indicating that the vowel was completely dissimilar to the chosen category and 5 indicating a very close match. The task included one practice block and eight test blocks, each consisting of 10 tokens. For each participant, the order of the blocks and the presentation of tokens within each block were randomized.

## 3. Results

### 3.1. Assimilation Types

A 70% categorization criterion was applied to determine whether an L2 English stimulus is categorized (Bundgaard-Nielsen et al., 2011; Fabra & Tyler, 2023). If one single native label category is assigned for more than 70% of all identification responses for a certain stimulus, the stimulus was considered categorized; otherwise, it was labeled uncategorized. When a particular contrast involved two members categorized as the same native phoneme, their category goodness ratings were analyzed using *t*-tests to determine whether the contrast was assimilated as an SC contrast with the same ratings or a CG contrast with different ratings (*p* < 0.05) (Fenwick et al., 2017; Nam et al., 2021; Tyler et al., 2014). For an uncategorized phone, one-sample *t*-tests were performed to examine the mean identification percentage of each L1 phoneme selected by Chinese listeners against a chance score of 16.67% to determine the assimilation types of uncategorized phones, i.e., focalized, clustered, and dispersed assimilations (Faris et al., 2016, 2018; Fenwick et al., 2017; Nam et al., 2021).

Table 1 presents the mean percentages of the identification responses and the category goodness ratings for the target English vowels in Chinese listeners. Percentages indicate how often each Chinese vowel was selected to identify the given English vowel across listeners.

As shown in Table 1, for English /i-ɪ/, Chinese listeners categorized /i/ as Chinese /i/ (91.8%) with a rating of 4.0 but they did not categorize /ɪ/, mostly dividing their responses between Chinese /i/ (60.9%) and /ɤ/ (26.6%). Thus, /i-ɪ/ yielded UC assimilation. English /e/, /ɛ/, and /æ/ were not reliably interpreted as a single Chinese category. For /e/, identification responses were spread across Chinese /i/ (45.3%), /ɤ/ (37.9%), and /ɑ/ (14.5%). For /ɛ/, identification responses were spread across Chinese /ɑ/ (48.0%), /ɤ/ (33.2%), and /i/ (14.8%). For /æ/, identification responses were spread across Chinese /ɑ/ (44.5%), /ɤ/ (37.5%), and /i/ (16.4%). Thus, both /e-ɛ/ and /æ-ɛ/ exhibited UU assimilations. For English /ɑ-ɔ/, Chinese listeners categorized both /ɑ/ (92.6%) and /ɔ/ (72.3%) as Chinese /ɑ/ with ratings of 4.0 and 3.6, respectively, yielding CG assimilation (*t*(3.19) = 361.93, *p* = 0.002). Chinese listeners failed to reach the categorization criterion for English /ʌ/, assigning their responses to Chinese /ɑ/ (58.2%), /ɤ/ (23.4%), /u/ (9.4%), and /o/ (8.2%). Thus, /ɔ-ʌ/ revealed UC assimilation. For English /u-ʊ/, Chinese listeners reliably categorized /u/ as Chinese /u/ (88.3%), whereas they did not categorize /ʊ/, assigning their responses to Chinese /u/ (55.1%), /ɑ/ (24.6%), /o/ (10.9%), and /ɤ/ (8.2%), yielding UC assimilation.

The uncategorized vowels /ɪ, e, ɛ, æ, ʌ, ʊ/ were submitted to one-sample *t*-tests to determine whether they were assimilated as focalized, clustered, or dispersed responses. English /ɪ/ showed focalized assimilation to Chinese /i/ (*t*(31) = 7.18, *p* < 0.0001), with the contrast /i-ɪ/ forming a UC-C assimilation with complete overlap on Chinese /i/. English /ʌ/ also showed focalized assimilation to Chinese /ɑ/ (*t*(31) = 6.50, *p* < 0.0001), with the contrast /ɔ-ʌ/ yielding a UC-C assimilation with complete overlap on Chinese /ɑ/. English /e, ɛ, æ/ were assimilated as clustered: /e/ was mapped to Chinese /ɤ/ (*t*(31) = 5.83, *p* < 0.0001) and /i/ (*t*(31) = 2.45, *p* = 0.021); /ɛ/ to /ɑ/ (*t*(31) = 4.92, *p* < 0.0001) and /ɤ/ (*t*(31) = 2.98, *p* = 0.006); and /æ/ to /ɑ/ (*t*(31) = 3.83, *p* = 0.001) and /ɤ/ (*t*(31) = 3.22, *p* = 0.003). Thus, the contrast /e-ɛ/ yielded a UU-P assimilation with partial overlap on Chinese /ɤ/, while /æ-ɛ/ yielded a UU-C assimilation with complete overlap on Chinese /ɑ/ and /ɤ/. English /ʊ/ exhibited clustered assimilation to Chinese /ɑ/ (*t*(31) = 8.98, *p* < 0.0001) and /u/ (*t*(31) = 8.50, *p* < 0.0001), resulting in a UC-P assimilation for the contrast /u-ʊ/ with partial overlap on /u/.

Based on the assimilation data, discrimination performance for the English vowel contrasts was predicted within the PAM-L2 framework (Best & Tyler, 2007; Faris et al., 2016, 2018). UC-P, UU-P, and CG contrasts were expected to be more accurately discriminated than UC-C and UU-C contrasts. Accordingly, the expected discrimination pattern is: (UU-P /e-ɛ/, UC-P /u-ʊ/, and CG /ɑ-ɔ/) > (UC-C /i-ɪ/, UC-C /ɔ-ʌ/, and UU-C /æ-ɛ/).

### 3.2. Dsicrimiantion Performance

**Figure 1:**
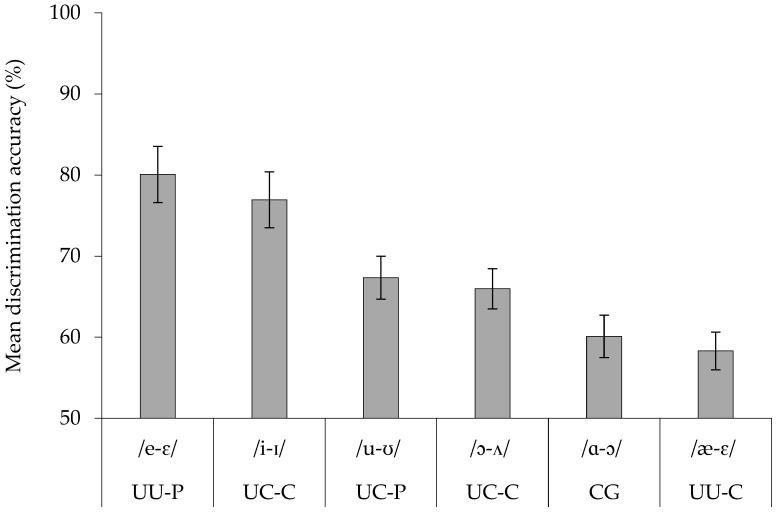
Mean discrimination accuracy for each English vowel contrast in Chinese listeners, with corresponding PAM assimilation types (UU-P, UC-C, CG) indicated. Error bars represent ±1 standard error.

For each participant, performance across all AXB pairs was calculated for each contrast. Three contrasts (/i-ɪ/, /e-ɛ/, /æ-ɛ/) showed significant deviations from normality (*W* ≤ 0.92, *p* ≤ 0.02), while the remaining three (/ɑ-ɔ/, /ɔ-ʌ/, /u-ʊ/) did not (*W* ≥ 0.95, *p* ≥ 0.20). Thus, discrimination data were submitted to a non-parametric Friedman’s ANOVA. The analysis revealed a significant difference in discrimination accuracy across the six vowel contrasts (χ^2^(5) = 69.99, *p* < 0.0001). Pairwise Wilcoxon signed-rank tests with Holm correction revealed that /e-ɛ/ and /i-ɪ/ did not differ significantly (*Z* = 1.62, *p* = 0.10). Discrimination accuracy for /i-ɪ/ was significantly higher than for all other contrasts (*Z* ≥ 3.27, *p* ≤ 0.001). /u-ʊ/ and /ɔ-ʌ/ did not differ (*Z* = 0.97, *p* = 0.33), but /ɔ-ʌ/ was discriminated more accurately than /ɑ-ɔ/ (*Z* = 2.76, *p* = 0.006). /ɑ-ɔ/ and /æ-ɛ/ did not differ (*Z* = 1.13, *p* = 0.26). These pairwise comparisons are summarized as follows: UU-P /e-ɛ/ (80.1%) and UC-C /i-ɪ/ (77.0%) > UC-P /u-ʊ/ (67.3%) and UC-C /ɔ-ʌ/ (66.0%) > CG /ɑ-ɔ/ (60.1%) and UU-C /æ-ɛ/ (58.3%). Figure 1 illustrates the mean discrimination accuracy for each contrast by Chinese listeners.

To better capture patterns underlying variability in discrimination performance—particularly within contrasts of the same assimilation type—overlap scores were computed for each English vowel contrast. Table 2 summarizes the identification patterns, discrimination performance, and overlap scores for the contrasts tested. The table also presents the most frequent and second most frequent native vowel categories selected to identify each English vowel, both of which were identified at above-chance levels. The overall overlap score represents the sum of the lower (or equal) assimilation percentages across all shared native categories used to identify both vowels in each contrast.

## 4. Discussion

This study examined how assimilation types shape variability in discrimination accuracy with a focus on whether such variability can be explained through the lens of assimilation overlap within the PAM-L2 perspective. Chinese listeners were tested with six English vowel contrasts. To better understand how the degree of overlap was associated with discrimination performance, overlap scores were computed for each contrast. All contrasts were assimilated as three UC and two UU contrasts with one exception (a CG contrast), which allowed for a focused examination of how differences in assimilation overlap may influence discrimination accuracy, particularly within the same types of assimilation.

### 4.1. Assimilation Overlap Affects Discriminability of L2 Vowel Contrasts

According to extended PAM-L2 predictions, partially overlapping UC or UU contrasts are expected to yield better discrimination than completely overlapping ones, with CG contrasts typically comparable to the latter. Chinese listeners assimilated /u-ʊ/ as UC-P, /ɔ-ʌ/ as UC-C, /æ-ɛ/ and /u-ʊ/ as UU-C, /e-ɛ/ as UU-P, and /ɑ-ɔ/ as CG. These patterns are generally consistent with the predictions based on Sun and van Heuven (2007) for contrasts expected to overlap, such as /i-ɪ/ and /ɔ-ʌ/, and they are also compatible with the CG /ɑ-ɔ/ which entails overlap because both vowels are assimilated to the same L1 category. In this respect, the prediction is consistent with the observed pattern. In contrast, /e-ɛ/ and /u-ʊ/ did not exhibit the predicted non-overlap. For example, Sun and van Heuven (2007) reported that Chinese listeners categorized English /u/ as Chinese /u/ (91%), while /ʊ/ was assimilated mainly to /oʊ/ (63%) and /ɑʊ/ (13%), yielding a non-overlapping pattern. Further cross-study variability comes from Lai (2010). In that study, both high- and low-proficiency groups consistently assimilated English /ɑ, ɔ, ʌ/ to Chinese /ɑ, o, ɑ/ in more than 70% of responses. This indicates that /ɑ-ɔ/ and /ɔ-ʌ/ showed no overlap in Lai’s data, unlike the overlap observed in the present study. Such differences suggest that assimilation patterns can vary across studies and may partially reflect differences in the number of response options (Lai, 2010, p. 9; Sun & van Heuven, 2007, p. 14; present study, p. 6).

With respect to discrimination, within the UU contrasts, discrimination accuracy was significantly higher for /e-ɛ/ (partial overlap) than for /æ-ɛ/ (complete overlap), consistent with PAM-L2 predictions (Best & Tyler, 2007; Faris et al., 2016, 2018). Within the UC contrasts, /i-ɪ/ (complete overlap) was discriminated more accurately than /u-ʊ/ (partial overlap) and /ɔ-ʌ/ (complete overlap), with no difference between the latter two. Discrimination of CG /ɑ-ɔ/ was similar to UU-C /æ-ɛ/ but lower than UC-C /i-ɪ/ and /ɔ-ʌ/. An overall comparison revealed that UC-P and UU-P did not consistently outperform UC-C, UU-C, and CG, providing partial support for PAM-L2 predictions. However, notable differences were also observed, both across contrasts and within the same overlap type.

To provide a more fine-grained account of the relationship between assimilation patterns and discrimination accuracy, following Levy (2009), overlap scores were derived from the assimilation data to quantify the extent of shared L1 response categories (Table 2). The UU-C contrast /æ-ɛ/ had the highest overlap score (94.1%) and yielded the lowest discrimination accuracy (58.3%). The CG contrast /ɑ-ɔ/, with the second-highest overlap score (78.1%), showed similarly low accuracy (60.1%). Despite the difference in overlap scores, there was no significant difference in discrimination accuracy between these two contrasts. These results align with the expectation that greater overlap reduces discriminability, but this relationship did not hold consistently across all contrasts. UC-P /u-ʊ/, which had the lowest overlap score (62.1%), was discriminated at a similar level to UC-C /ɔ-ʌ/, which had a higher overlap score (69.5%), with both showing mid-level discrimination accuracy. UU-P /e-ɛ/ had an overlap score of 63.7% and yielded the highest discrimination accuracy. UC-C /i-ɪ/ showed a similar pattern, with an overlap score of 68.8% and comparably high accuracy. Notably, UU-P /e-ɛ/ and UC-P /u-ʊ/ had comparable overlap scores, as did UC-C /i-ɪ/ and UC-C /ɔ-ʌ/, but each pair showed clear differences in discrimination accuracy. These findings suggest that, while overlap scores are associated with discrimination performance in some contrasts, particularly those with relatively high overlap, they do not serve as a consistent predictor.

A closer examination of the category overlap patterns in Table 2 reveals that for UU-P /e-ɛ/, Chinese /i/ and /ɤ/ were the most and second most frequently selected categories to identify the uncategorized /e/, while /ɑ/ and /ɤ/ were the most and second most frequent responses for /ɛ/. Thus, the only overlap among above-chance response categories occurred between the second-most frequent labels (/ɤ/ for both vowels) and served as the primary source of overlap. Importantly, the dominant response categories (/i/ for /e/ and /ɑ/ for /ɛ/) were entirely distinct and did not overlap. In this sense, the primary overlap occurred between a pair of secondary categories, unlike other contrasts. In the remaining UC and UU contrasts, the primary overlap was found between the most frequently selected above-chance L1 categories for both vowels (e.g., UC-P /u-ʊ/), or, in one case (UU-C /æ-ɛ/), the two vowels shared both their first- and second-most frequently selected above-chance categories.

This pattern observed for /e-ɛ/ became more distinctive when considering the overlap scores based on dominant first and/or second categories; /e-ɛ/ involved overlap (33.2%) only from the second-most frequent category with no overlap between the dominant categories (Table 1). Compared to other UU or UC contrasts, this suggests a less convergent and more diffuse pattern of perceptual overlap. /u-ʊ/ (UC-P) and /ɔ-ʌ/ (UC-C) showed convergence at the dominant category level (55.1% and 58.2%, respectively), while /æ-ɛ/ (UU-C) exhibited convergence in both dominant (/ɑ/) and secondary (/ɤ/) categories (77.7%). /ɑ-ɔ/ (CG) naturally exhibited strong dominant-category convergence, with its shared category exceeding the 70% criterion. In contrast, /e-ɛ/ lacked such dominant-category convergence, which may have facilitated perceptual separation and contributed to its relatively high discrimination accuracy despite an overall overlap score that appears relatively large.

### 4.2. Goodness Rating Difference Affect Discriminability of L2 Vowel Contrasts

UC-C /i-ɪ/ was discriminated more accurately than both UC-P /u-ʊ/ and UC-C /ɔ-ʌ/, despite having a higher overlap score than the former and a comparable score to the latter. Both categorized /i/ and uncategorized /ɪ/ were mapped to Chinese /i/ with complete overlap, but their goodness ratings showed a large difference: /i/ and /ɪ/ were rated 4.0 and 3.1, respectively (Cohen’s d = 0.77). This pattern suggests a perceptual disparity between the two vowels, with /i/ functioning as a more prototypical exemplar of the shared L1 category. Thus, UC-C /i-ɪ/ appeared to be perceived as a within-category contrast with a large goodness difference, which likely provided cues supporting discrimination.

No such meaningful rating difference was observed for UC-C /ɔ-ʌ/ (Cohen’s d = 0.07), where both vowels were mapped to the same L1 category. This absence of a meaningful goodness difference, combined with the complete overlap, likely limited listeners’ ability to discriminate the contrast. Furthermore, CG /ɑ-ɔ/ involved a small difference in goodness ratings (Cohen’s d = 0.32) and yielded reduced discrimination accuracy, supporting the PAM-based view that within-category perception depends on goodness differences (Best, 1994, 1995; Best & Tyler, 2007).

## 5. Conclusions

This study examined how assimilation types, particularly assimilation overlap, relate to variability in L2 vowel discrimination within the PAM-L2 framework (Best & Tyler, 2007; Faris et al., 2016, 2018). While some findings aligned with PAM-L2 predictions, the results also reveal how overlap patterns and category goodness differences can influence L2 vowel discrimination. Discrimination appeared to be facilitated when dominant L1 response categories did not overlap, even when secondary categories overlapped and large differences in category goodness aided perception of completely overlapping contrasts. These findings point to a potential interaction between assimilation overlap and category goodness in within-category perception. The findings also suggest implications for L2 teaching instruction. The difficulty Chinese listeners showed with vowel contrasts involving assimilation overlap (e.g., /æ-ɛ/, /ɑ-ɔ/) points to the need for targeted perception training on these contrasts. Such training may help L2 learners establish distinctive perceptual categories within the English vowel system and thereby support more accurate perception.

However, there are several limitations in this study. The stimuli were produced by only one speaker, restricting talker variability and potentially limiting generalizability. In addition, listeners were primarily advanced Chinese learners of Korean and their experience with Korean may have influenced their perception of English vowels. A meaningful direction for future research will be to test Chinese listeners with no prior knowledge of Korean. Importantly, since there was no instance of non-overlapping assimilation, the analysis of overlap scores and goodness ratings provides only exploratory evidence and should be interpreted with caution. Therefore, additional data are needed to clarify how overlap and rating differences shape L2 vowel perception. Such data would also allow for a more comprehensive evaluation of the PAM-L2 account of the overlap-discrimination relationship.

## Figures and Tables

**Table 1:** Mean percent assimilation and goodness ratings (in parentheses) of English vowels in terms of Chinese vowel categories by Chinese listeners. Goodness ratings were based on a 5-point scale, ranging from 1 (very dissimilar) to 5 (identical).

		Chinese Vowels					
		i	y	ɑ	ɤ	o	u
English Vowels	i	**91.8 (4.0)** ^1^		1.2 (2.3)	5.9 (4.0)	0.8 (2.5)	
	ɪ	***60.9 (3.1)*** ^2^	1.2 (3.3)	8.6 (1.9)	26.6 (3.2)	1.2 (3.0)	
	e	***45.3 (2.9)***	2.0 (2.6)	14.5 (1.5)	***37.9 (2.8)***		
	ɛ	14.8 (2.3)	1.2 (2.7)	***48.0 (2.2)***	***33.2 (2.7)***	1.6 (3.0)	1.2 (2.0)
	æ	16.4 (2.0)	0.8 (2.5)	***44.5 (2.2)***	***37.5 (2.6)***	0.4 (3.0)	0.4 (3.0)
	ɑ			**92.6 (4.0)**	1.6 (3.0)	2.7 (4.0)	2.0 (3.4)
	ɔ	0.4 (3.0)		**72.3 (3.6)**	1.6 (3.3)	24.2 (3.5)	1.6 (4.0)
	ʌ			***58.2 (3.5)***	23.4 (3.6)	8.2 (3.3)	9.4 (3.5)
	ʊ			***24.6 (3.9)***	8.2 (3.7)	10.9 (3.1)	***55.1 (4.0)***
	u		4.7 (4.1)		0.4 (4.0)	6.6 (3.0)	**88.3 (4.2)**

^1^ Boldface indicates that the English vowel was categorized (>70%). ^2^ Boldface italics indicate that the English vowel was not categorized but was identified above chance (16.67%).

**Table 2:** Summary of identification patterns, assimilation types, discrimination accuracy, and overall overlap scores for English vowel contrasts in Chinese listeners. Overall overlap score represents the percentage of shared native categories between the two vowels for each contrast, calculated from the most frequent responses and/or from the second most frequent responses. Gray shading indicates overlap in Chinese labels used to identify the two English vowels for each contrast. “C” and “U” denote categorized and uncategorized vowels, respectively.

English vowel contrasts	/e-ɛ/		/i-ɪ/		/u-ʊ/		/ɔ-ʌ/		/ɑ-ɔ/		/æ-ɛ/	
Categorization status	/e/	/ɛ/	/i/	/ɪ/	/u/	/ʊ/	/ɔ/	/ʌ/	/ɑ/	/ɔ/	/æ/	/ɛ/
	U	U	C	U	C	U	C	U	C	C	U	U
Native response choices *	/i/	/ɑ/	/i/	/i/	/u/	/u/	/ɑ/	/ɑ/	ɑ	ɑ	/ɑ/	/ɑ/
	/ɤ/	/ɤ/				/ɑ/					/ɤ/	/ɤ/
Assimilation type	UU-P		UC-C		UC-P		UC-C		CG		UU-C	
Discrimination accuracy (%)	80.1		77.0		67.3		66.0		60.1		58.3	
Overall overlap score (%)	63.7		68.8		62.1		69.5		78.1		94.1	

* The top and bottom rows indicate the most and second most frequently selected native vowel categories, respectively, each identified at above-chance levels.

## Data Availability

Not applicable.
